# Endoscopic ultrasound-guided sampling of solid pancreatic masses: 22-gauge aspiration versus 25-gauge biopsy needles

**DOI:** 10.1186/s12876-015-0352-9

**Published:** 2015-09-29

**Authors:** Min Jae Yang, Hyunee Yim, Jae Chul Hwang, Dakeun Lee, Young Bae Kim, Sun Gyo Lim, Soon Sun Kim, Joon Koo Kang, Byung Moo Yoo, Jin Hong Kim

**Affiliations:** 1 Department of Gastroenterology, Ajou University School of Medicine, San-5, Woncheon-dong, Yongtong-gu, 443-721 Suwon, Korea; 2 Department of Pathology, Ajou University School of Medicine, San-5, Woncheon-dong, Yongtong-gu, 443-721 Suwon, Korea

**Keywords:** *Endoscopic ultrasound*, *EUS-guided fine needle aspiration*, *Pancreatic mass*

## Abstract

**Background:**

Biopsy needles have recently been developed to obtain both cytological and histological specimens during endoscopic ultrasound (EUS). We conducted this study to compare 22-gauge (G) fine needle aspiration (FNA) needles, which have been the most frequently used, and new 25G fine needle biopsy (FNB) needles for EUS-guided sampling of solid pancreatic masses.

**Methods:**

We conducted a retrospective cohort study of all EUS-guided sampling performed between June 2010 and October 2013. During the study period, 76 patients with pancreatic masses underwent EUS-guided sampling with a 22G FNA needle (*n* = 38) or a 25G FNB needle (*n* = 38) for diagnosis. An on-site cytopathologist was not present during the procedure. Technical success, the number of needle passes, cytological diagnostic accuracy, cytological sample quality (conventional smear and liquid-based preparation), histological diagnostic accuracy, and complications were reviewed and compared.

**Results:**

There were no significant differences in technical success (100 % for both), the mean number of needle passes (5.05 vs. 5.55, *P* = 0.132), or complications (0 % for both) between the 22G FNA group and the 25G FNB group. The 22G FNA and 25G FNB groups exhibited comparable outcomes with respect to cytological diagnostic accuracy (97.4 % vs. 89.5 %, *P* = 0.358) and histological diagnostic accuracy (34.2 % vs. 52.6 %, *P* = 0.105). In the cytological sample quality analysis, the 25G FNB group exhibited higher scores for the amount of diagnostic cellular material present (22G FNA: 0.92 vs. 25G FNB: 1.32, *P* = 0.030) and the retention of appropriate architecture (22G FNA: 0.97 vs. 25G FNB: 1.42, *P* = 0.010) in the liquid-based preparation. The 25G FNB group showed a better histological diagnostic yield for specific tumor discrimination compared with the 22G FNA group (60 % vs. 32.4 %, *P* = 0.018).

**Conclusions:**

Use of the 25G FNB needle was technically feasible, safe, efficient, and comparable to use of the standard 22G FNA needle in patients with solid pancreatic masses in the absence of an on-site cytopathologist. The cytological sample quality in the liquid-based preparation and the histological diagnostic yield for specific tumor discrimination of EUS-guided sampling using a 25G FNB needle were significantly higher than those using a 22G FNA needle.

## Background

Patients with pancreatic cancer have a dismal prognosis, with a 5-year survival rate of approximately 5 %. Therefore, rapid and correct diagnosis of a pancreatic mass is required to guide subsequent patient management. Endoscopic ultrasound (EUS)-guided fine needle aspiration (FNA) is the current standard method for obtaining tissue for the diagnosis of pancreatic masses [1, 2]. The reported results of pancreatic EUS-FNA vary in the range of 64–95 % for sensitivity, 75–100 % for specificity, and 78–95 % for diagnostic accuracy [3–5]. Several factors can affect the results of EUS-FNA, such as the experience of the endosonographer, the position of the endoscope, the diameter of the needle, the number of passes, and the presence of an on-site cytopathologist [6–11]. Furthermore, core biopsy specimens for assessing architectural features may be essential for diagnosing certain neoplasms, such as lymphomas and stromal cell tumors [12–14]. On-site cytological evaluation of EUS-FNA specimens by a cytopathologist has also been reported to increase the diagnostic yield [7, 15]. However, cost and staffing limitations frequently limit the availability of an on-site cytopathologist at many centers.

The 22-gauge (G) FNA needle has been the most commonly used for EUS-FNA of pancreatic masses [9, 11]. Both 19G and 22G fine needle biopsy (FNB) needles with a reverse-bevel to trap core specimens were developed to obtain core biopsy specimens. The results have indicated high yields of core specimens and fewer passes to provide adequate samples [16, 17]. However, a 25G FNA needle may be less traumatic and easier to manipulate when the tip of the echoendoscope is completely angulated. The aspirate obtained with a 25G FNA needle may also be less hemorrhagic and more adequate for interpretation by cytologists [18]. In fact, a new 25G FNB needle with a reverse-bevel-sided hole to obtain core specimens for histological evaluation is available. However, there is little data to support the use of the 25G FNB needle for EUS-guided sampling of pancreatic masses. The aim of the present study was to evaluate the feasibility, safety, and diagnostic adequacy of the 25G FNB needle in patients with solid pancreatic masses and to compare its performance with that of the widely used standard 22G FNA needle without on-site cytopathological examination.

## Methods

### Patients

We conducted a retrospective cohort study of all EUS-guided sampling performed between June 2010 and October 2013 at a single tertiary referral hospital (Ajou University Hospital, Suwon, Korea). The EUS database at our institution was searched for prospectively collected data on patients with solid pancreatic mass lesions who underwent EUS-guided sampling. During the study period, we performed 108 consecutive cases of EUS-guided sampling including 76 cases of solid pancreatic lesions, 16 cases of gastric subepithelial tumors, 12 cases of lymph nodes, and 4 cases of esophageal subepithelial tumors. The 25G FNB needle is a smaller-gauge needle and has a side bevel for the acquisition of core specimens; we believed that its smaller size would allow greater flexibility in puncturing tissue when the tip of the echoendoscope was completely angulated and that the side bevel could obtain core specimens, which could improve the diagnostic yield of targeted lesions. Thus, since the 25G FNB needle system with reverse-bevel technology for the acquisition of core specimens was introduced at this hospital (June 2012), we have used the needle exclusively for all EUS-guided sampling. In total, 38 patients were included in the 22G FNA group, and 38 patients were included in the 25G FNB group. The medical records of the patients were reviewed using a standardized data-entry form that included patient demographics, clinical findings, procedural complications, follow-up, and pathological findings. The results of EUS-guided sampling were confirmed using the following criteria: surgical histopathology, clinical follow-up for a minimum of 6 months, and/or the results of other diagnostic tests. This study was approved by the Institutional Review Board of the Ajou University Hospital (AJIRB-MED-MDB-13-327), and informed consent was obtained from all patients for whom EUS-guided sampling was performed.

### EUS-guided sampling technique

**Fig. 1:**
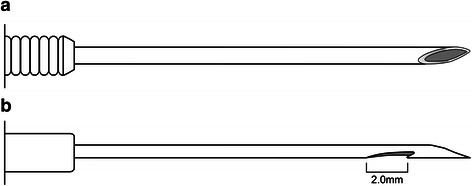
Schematic representation of the needles used in this study. **a** 22-gauge fine needle aspiration needle. **b** 25-gauge fine needle biopsy needle with a reverse bevel for tissue acquisition

EUS-guided sampling was performed using a linear array echoendoscope (GF-UCT 2000; Olympus Optical, Tokyo, Japan) in hospitalized patients. Each patient was sedated with standard doses of midazolam, propofol, and meperidine. After the optimal puncture site was determined, a puncture was made using a 22G FNA needle (Echotip Ultra; Cook Ireland Ltd., Limerick, Ireland) or a 25G FNB needle (Echotip ProCore; Cook Ireland Ltd., Limerick, Ireland) under EUS image guidance (Fig. 1). All pancreatic head and uncinate process masses were approached via the duodenum, and all pancreatic body and tail masses were approached via the stomach. After the lesion was punctured, the stylet was removed, and suction was applied using a 10-mL syringe. On every pass, the needle was moved to and fro within the lesion 10–15 times in a fanning manner.

An on-site cytopathologist was not present during EUS-guided sampling. The needle stylet was used to gradually push the tissue material out of the needle. The materials obtained on the first pass of EUS-guided sampling were mounted onto slides for conventional smear (CS) with alcohol fixation, and the materials obtained from the following pass of EUS-guided sampling were placed into an ethanol-based preservative (CytoRichRed™; Becton-Dickinson Diagnostics, Burlington, NC, USA) for SurePath™ (Becton-Dickinson Diagnostics, Burlington, NC, USA), a liquid-based preparation (LBP). If the materials for CS or LBP that were assessed macroscopically by the endosonographer were insufficient, more passes of EUS-guided sampling for CS or LBP were performed. After we obtained the material for CS and LBP, additional passes were performed to obtain histological specimens. The extruded material was placed onto glass slides for primary inspection by conventional smear or histological examination. In liquid-based preparations, the material in the needle was directly expelled into an ethanol-based preservative solution for SurePath™. The aspirated material for histological examination was assessed macroscopically by the endosonographer. Macroscopically sufficient material for histological evaluation was defined as one or more small-core biopsy cylinders. If one or more small-core biopsy cylinders were acquired, the tissue material was placed in formalin solution. The number of needle passes to obtain a histological specimen was limited to 2 to prevent procedure-related complications.

### Preparation of specimens and sample quality

Papanicolaou stain was applied to both CS and LBP slides. Histological diagnosis of biopsy specimens was carried out for hematoxylin-eosin stained specimens. We performed immunochemical staining of LBP slides or histological specimens for synaptophysin, chromogranin, MIB-1, beta-catenin, CD 10, CD 56, CD 99, and PR in selected cases that were suspected to contain pseudopapillary tumors, neuroendocrine tumors or metastatic tumors.

For the assessment of cytological sample quality, the scoring system reported by Mair *et al.* [19] was used (Table 1). The two techniques for EUS-guided sampling were compared based on the following parameters: (1) the amount of diagnostic cellular material present, (2) the retention of appropriate architecture and cellular arrangement, (3) the degree of cellular degeneration, (4) the degree of cellular trauma, and (5) the volume of obscuring background blood or clot. The assessment of cytological sample quality using this scoring system is not daily clinical practice in our pathological department, and it was performed retrospectively only for this study by one cytopathologist (HY) who was blinded to the type of the needle and the results from the other preparation technique for every case. Two pathologists (YBK and DL) participated in the histological examinations, which were performed at the time of diagnosis, and we retrospectively reviewed the pathologic reports for histological assessment.

**Table 1:** Scoring described by Mair *et al.* [19]

Parameter	Description	Score
Amount of cellular material	Minimal to absent; diagnosis not possible	0
	Sufficient for cytodiagnosis	1
	Abundant; diagnosis simple	2
Retention of appropriate architecture	Minimal to absent; nondiagnostic	0
	Moderate; some preservation	1
	Excellent; diagnosis obvious	2
Degree of cellular degeneration	Marked; diagnosis impossible	0
	Moderate; diagnosis possible	1
	Minimal; good preservation; diagnosis easy	2
Degree of cellular trauma	Marked; diagnosis not possible	0
	Moderate; diagnosis possible	1
	Minimal; diagnosis obvious	2
Background blood or clot	Large amount; great compromise to diagnosis	0
	Moderate amount; diagnosis possible	1
	Minimal; diagnosis easy; specimen of “textbook” quality	2

### Outcome parameters

The primary outcome parameter was diagnostic accuracy, which was defined as the ratio between the sum of true positive and true negative values divided by the total number of lesions for diagnosing neoplasia versus benign (non-neoplastic) conditions. Malignant and suspicious lesions (diagnosed by cytopathology on samples obtained using EUS-guided sampling) that were eventually diagnosed as malignant were considered to be true positives. We did not include atypical cytopathology results as positives for malignancy. The secondary outcome measures were technical failure, the number of needle passes, complications, cytological sample quality, and the ability to provide a specific tissue diagnosis, *e.g.*, adenocarcinoma, metastasis, neuroendocrine tumor, or pseudopapillary tumor, for cases of neoplasia. Early and late complications were defined as any EUS-guided sampling-related complication within 30 days and after 30 days of the procedure, respectively. Serum amylase levels, complete blood counts, and abdominal radiographs were checked on the day following EUS-guided sampling to monitor complications such as bleeding, perforation, and acute pancreatitis. All patients were instructed to let us know if any symptoms suggestive of a complication were noted. Additional information regarding current status was obtained via direct contact with the referring physician.

### Statistical analysis

Statistical analysis was performed using the chi-square test or Fisher’s exact test for categorical parameters and Student’s *t*-test for continuous variables. Analyses were performed using SPSS for Windows (version 18.0; SPSS Inc., Chicago, IL, USA), with quantitative data presented as the mean ± standard deviation. Statistical significance was set at a *P* value of < 0.05.

## Results

Demographic data for the 76 study patients are shown in Table 2. There was no significant difference between the 22G FNA group and the 25G FNB group with regard to the male-to-female ratio, age, mass size, mass location, or final diagnosis. Surgical histopathology was available for 10 cases in the 22G FNA group and three cases in the 25G FNB group. The residual cases were corroborated by clinical follow-up. The median follow-up was 239 days in the 22G FNA group and 253 days in the 25G FNB group. After EUS-guided sampling, 39/64 patients with pancreatic adenocarcinoma or metastasis received chemoradiation, seven underwent surgical resection, and 18 were managed conservatively. Additionally, four patients with neuroendocrine tumors and two patients with pseudopapillary tumors underwent surgical resection. None of the six patients with chronic pancreatitis exhibited disease progression during clinical follow-up.

**Table 2:** Baseline patient and tumor characteristics

	22-gauge FNA (*n* = 38)	25-gauge FNB (*n* = 38)	*P* value
Sex (male/female)	17/21	18/20	0.818
Age (years)	61.8 ± 11.4	63.0 ± 12.6	0.669
Size of mass on EUS (mm)	34.1 ± 12.6	33.8 ± 16.3	0.931
Tumor location, no. (%)			0.359
Head/uncinate	21 (55.3)	17 (44.7)	
Body/tail	17 (44.7)	21 (55.3)	
Final diagnosis, no. (%)			0.187
Adenocarcinoma	33 (86.8)	29 (76.3)	
Metastasis	0 (0)	2 (5.3)	
Neuroendocrine tumor	3 (7.9)	1 (2.6)	
Pseudopapillary tumor	1 (2.6)	1 (2.6)	
Chronic pancreatitis	1 (2.6)	5 (13.2)	

*EUS* endoscopic ultrasound, *FNA* fine needle aspiration, *FNB* fine needle biopsy

Continuous variables are expressed as the mean ± standard deviation

There was no significant difference in the access route, the technical success rate, cytological diagnostic accuracy, or histological diagnostic accuracy between the two groups (Table 3). The technical success rate was 100 % in both groups. The overall diagnostic accuracy regarding pancreatic masses was consistent with the cytological diagnostic accuracy in both groups. In particular, the cytological diagnostic accuracy was 97.4 % (37/38) in the 22G FNA group and 89.5 % (34/38) in the 25G FNB group (*P* = 0.358). There was no significant difference in the diagnostic accuracy between CS and LBP within the groups (22G FNA: 89.5 % [34/38] vs. 76.3 % [29/38], *P* = 0.128; 25G FNB: 84.2 % [32/38] vs. 84.2 % [32/38], *P* = 1.000). In the cytological diagnosis of the 22G FNA and 25G FNB groups, CS complemented LBP in eight cases and two cases, respectively, and LBP complemented CS in three cases and two cases, respectively. In the sample quality analysis, the two sampling techniques with CS demonstrated similar scores for the amount of diagnostic cellular material present, the retention of appropriate architecture and cellular arrangement, the degree of cellular degeneration, the degree of cellular trauma, and the volume of obscuring background blood or clot (Table 4). For LBP, the 25G FNB group exhibited higher scores for the amount of diagnostic cellular material present (22G FNA: 0.92 vs. 25G FNB: 1.32, *P* = 0.030) and the retention of appropriate architecture (22G FNA: 0.97 vs. 25G FNB: 1.42, *P* = 0.010) (Table 5). The rate of acquiring sufficient material, as assessed macroscopically by the endosonographer for histological evaluation, was not significantly different between groups (22G FNA 60.5 % vs. 25G FNB 68.4 %, *P* = 0.472). The histological diagnostic accuracy regarding pancreatic masses was 34.2 % (13/38) in the 22G FNA group and 52.6 % (20/38) in the 25G FNB group (*P* = 0.105). The results for both cytology and histology in distinguishing tumor types are shown in Table 6. For cytology, there was no difference between the 22G FNA group and the 25G FNB group. For histology, the 25G FNB group demonstrated a better diagnostic yield for specific tumor discrimination compared with the 22G FNA group (20/33, 60.6 % vs. 12/37, 32.4 %; *P* = 0.018). There was no significant difference in the mean number of needle passes between the groups (22G FNA: 5.05 vs. 25G FNB: 5.55, *P* = 0.132) (Table 3). No early or late complications were identified in either group.

**Table 3:** Technical characteristics and outcomes of EUS guided sampling

	22-gauge FNA (*n* = 38)	25-gauge FNB (*n* = 38)	*P* value
Access route, no (%)			0.359
Transgastric	17 (44.7)	21 (55.3)	
Transduodenal	21 (55.3)	17 (44.7)	
Cytological diagnostic accuracy, no. (%)	37 (97.4)	34 (89.5)	0.358
Histological diagnostic accuracy, no. (%)	13 (34.2)	20 (52.6)	0.105
Failure to achieve diagnosis, no. (%)			
Total	1 (2.6)	4 (10.5)	0.358
Technical failure	0	0	
Diagnostic failure	1 (2.6)	4 (10.5)	0.358
No. of passes for diagnosis	5.05 ± 1.45	5.55 ± 1.41	0.132
Complication, no. (%)	0	0	

*EUS* endoscopic ultrasound, *FNA* fine needle aspiration, *FNB* fine needle biopsy

Continuous variables are expressed as the mean ± standard deviation

**Table 4:** Sample quality results for conventional smear^a^

	22-gauge FNA (*n* = 38)	25-gauge FNB (*n* = 38)	*P* value
Amount of cellular material	1.26 ± 0.72	1.55 ± 0.72	0.085
Retention of appropriate architecture	1.39 ± 0.68	1.63 ± 0.67	0.132
Degree of cellular degeneration	1.42 ± 0.60	1.58 ± 0.64	0.271
Degree of cellular trauma	1.26 ± 0.55	1.53 ± 0.65	0.061
Background blood or clot	1.29 ± 0.70	1.53 ± 0.73	0.150
Total score	6.63 ± 2.90	7.82 ± 3.14	0.092

*FNA* fine needle aspiration, *FNB* fine needle biopsy

Continuous variables are expressed as the mean ± standard deviation

^a^ The scoring system reported by Mair *et al.* [19] was used for the assessment of sample quality

**Table 5:** Sample quality results for liquid-based preparation^a^

	22-gauge FNA (*n* = 38)	25-gauge FNB (*n* = 38)	*P* value
Amount of cellular material	0.92 ± 0.78	1.32 ± 0.78	0.030
Retention of appropriate architecture	0.97 ± 0.75	1.42 ± 0.72	0.010
Degree of cellular degeneration	1.37 ± 0.75	1.61 ± 0.60	0.132
Degree of cellular trauma	1.26 ± 0.72	1.47 ± 0.60	0.173
Background blood or clot	1.11 ± 0.69	1.24 ± 0.71	0.416
Total score	5.63 ± 3.32	7.05 ± 3.00	0.054

*FNA* fine needle aspiration, *FNB* fine needle biopsy

Continuous variables are expressed as the mean ± standard deviation

^a^ The scoring system reported by Mair *et al.* [19] was used for the assessment of sample quality

**Table 6:** Proportion of cases in which smear, liquid-based preparation and histology correctly distinguished specific tumor types^a^

	22-gauge FNA			25-gauge FNB		
Specific diagnosis	CS	LBP	Histology	CS	LBP	Histology^b^
Adenocarcinoma	27/33	23/33	10/33	24/29	23/29	18/29
Metastasis	0/0	0/0	0/0	0/2	1/2	1/2
Neuroendocrine tumor	0/3	2/3	1/3	0/1	1/1	1/1
Pseudopapillary tumor	0/1	1/1	1/1	0/1	1/1	0/1
Total	27/37	23/37	12/37	24/33	26/33	20/33

*FNA* fine needle aspiration, *FNB* fine needle biopsy, *CS* conventional smear, *LBP* liquid-based preparation

^a^Only neoplastic cases are included

^b^The 25-gauge FNB group showed a better diagnostic yield in terms of specific tumor discrimination compared with the 22-gauge FNA group (*P* = 0.018)

## Discussion

In the current study, which compared the diagnostic yield and performance of the 25G FNB needle with the standard 22G FNA needle for the evaluation of pancreatic solid masses without on-site evaluation of specimens, the diagnostic yield, technical performance, and safety profile of the 25G FNB needle were comparable to those of the 22G FNA needle. Although our study failed to demonstrate the superiority of the 25G FNB needle over the 22G FNA needle in terms of the overall histological diagnostic yield, the 25G FNB group demonstrated a better histological diagnostic yield for specific tumor discrimination compared with the 22G FNA group.

Needle selection for EUS-guided sampling can be a complex process in clinical practice. The 22G FNA needle has been the most frequently used for EUS-FNA of pancreatic masses [9, 11], but the 25G FNA needle could be particularly useful for targeting lesions requiring extreme scope bending, as its smaller caliber and greater flexibility allow it to puncture tissue in hard pancreatic masses more easily [10]. This needle can also provide less bloody and less contaminated specimens, which may facilitate on-site interpretation [9, 10]. However, one meta-analysis reported no significant differences in accuracy, complication rates, the number of needle passes, or needle visibility when comparing 22G and 25G FNA needles [20]. In the present study, although there was no significant difference, there was a trend toward a higher score for background blood or clot in the samples from both the conventional smear and liquid-based preparations in the 25G FNB group compared with the 22G FNA group. Although cytological examination of EUS-FNA specimens enables us to detect malignancies, particular neoplasms such as lymphomas and gastrointestinal stromal tumors may be difficult to diagnose without histological specimens. Additionally, histology may be required for better, more specific characterization of pancreatic neoplasms other than adenocarcinomas [21, 22]. In fact, Möller *et al.* [22] reported that combining EUS-FNA cytology and histology with the 22G FNA needle significantly increased the sensitivity of malignancy diagnosis compared with cytology or histology alone. The sensitivity of histology alone was only 60 %, and the sensitivity of cytology alone was 68.1 %. Combining cytology and histology improved sensitivity to 82.9 %. In our study, the overall diagnostic accuracy regarding pancreatic masses was consistent with the cytological diagnostic accuracy in both groups. There was no improvement in diagnostic accuracy with the combination of cytology and histology. In the study by Möller *et al.* [22], core specimens were harvested for histological analysis first, and the remaining material was examined cytologically; however, in our study, samples for histological analysis were collected after tissue acquisition for cytological analysis. This different order may have caused the difference in the results. Recently, FNB needles of various sizes with a reverse-bevel-sided hole were developed to acquire core specimens for histological assessment. In a study of the 19G FNB needle by Iglesias-Garcia *et al.* [16], sample quality for histology was adequate in 45/47 pancreatic lesions (95.7 %), and correct diagnosis based solely on histology was provided in 42/47 (89.4 %), with two technical failures in the transduodenal approach. The needle emerged from the echoendoscope with difficulty in 18 % of cases, and this technical difficulty was experienced when the transduodenal approach was performed because of the rigidity induced by its 19G caliber and the curved position of the echoendoscope in the duodenum. In another study, Bang *et al.* [23] compared the performance of the 22G FNA needle and the 22G FNB needle in 56 patients with solid pancreatic masses. The specimens obtained were reviewed by an on-site cytopathologist to ascertain sample adequacy after each pass. In that study, there was no significant difference in the median number of needle passes (1 vs. 1, *P* = 0.21), technical failure (0 vs. 3.6 %, *P* = 1.0), the rates of diagnostic sufficiency (100 % vs. 89.3 %, *P* = 0.24), or the presence of diagnostic histological specimens (66.7 % vs. 80 %, *P* = 0.66) between the FNA and the FNB cohorts. Although the small-caliber 22G FNB needle obtained an adequate sample for cytological analysis, the quantity and quality of the acquired tissue appeared to be unsatisfactory for histological assessment. The histological diagnostic yield of the 22G FNA needle in our study (34.2 %) was lower than that reported by the previous study (66.7 %). There is one explanation that may contribute to this difference. In the study of Bang *et al.*, the specimens obtained with the 22G FNA needle underwent cell block analysis for histological assessment, and some studies have shown that cell block is a valid technique for performing histological assessments and can improve the diagnostic accuracy of smears [24–26]. In our study, the specimens for histological examination were placed in formalin solution, and the cell block technique was not used for histological assessment. In the present study, we observed no technical failure in either group, and there was no significant difference in cytological diagnostic accuracy between the groups. Although there was no statistically significant difference for CS, there was a trend toward higher scores for all categories in the sample quality analysis in the 25G FNB group compared with the 22G FNA group. Moreover, the 25G FNB group exhibited higher scores for the amount of diagnostic cellular material and the retention of appropriate architecture when LBP was used. In addition, there was a trend toward the 25G FNB needle exhibiting higher overall histological diagnostic accuracy than the 22G FNA needle (52.6 % vs. 34.2 %, *P* = 0.105), and the 25G FNB group showed a better histological diagnostic yield in specific tumor discrimination compared with the 22G FNA group (60.6 % vs. 32.4 %, *P* = 0.018). However, these results were unsatisfactory compared with those of previous studies, which used larger-gauge FNB needles [16, 23]. In the present study, samples for histological analysis were collected after tissue acquisition for cytological analysis, which was somewhat biased against histology. We performed EUS-guided sampling in inoperable solid pancreatic lesions and solid pancreatic lesions, in which it was difficult to differentiate between malignant and benign lesions in other diagnostic tests. Because cytology is more sensitive than histology alone for the diagnosis of pancreatic malignancies [27], we first obtained specimens for cytological examination. After the material for cytological examination was obtained, we restricted the number of needle passes for obtaining histological specimens to 2 to prevent procedure-related complications such as pancreatitis, bleeding, bile peritonitis or malignant seeding [28]. These factors may be related to the relatively low histological diagnostic yield compared with previous studies, which used larger-gauge FNB needles [16, 23]. A specimen obtained using a smaller-gauge needle may be less hemorrhagic and more adequate for cytological diagnosis. However, blood contamination does not decrease the histological diagnostic yield. Although we believe that the side bevel of the 25G FNB needle can allow cells to move into the needle more easily, larger-gauge FNB needles can offer a greater chance of obtaining an intact histological core. Prospective comparative studies are needed to evaluate the histological diagnostic yield of FNB needles of different sizes.

Previous studies recommended performing 5–7 passes of EUS-FNA for pancreatic masses [29–31]. In the present study, there was no significant difference in the mean number of passes required to make a diagnosis between the two groups (22G FNA: 5.05 vs. 25G FNB: 5.55, *P* = 0.132). Although we performed more passes to obtain a diagnosis with the FNA or FNB needle than in previous studies (mean number of passes for diagnosis: 1.28–2.78) [11, 22, 23], complications were not observed in either group.

Our patient group was small, and the study was limited by its retrospective nature. We divided the patients chronologically, according to the time period during which the needles were used, into two groups. Therefore, the bias-related improvements in the techniques of the operator may have been introduced. Another limitation of our study was the lack of surgical histopathology in many patients and the relatively short follow-up, during which all of our patients with pancreatic adenocarcinoma or metastasis exhibited disease progression. In contrast, none of the patients with chronic pancreatitis exhibited disease progression after a minimum of 6 months of follow-up. This follow-up period was adopted from similar studies [16, 23]. In the present study, it was also not possible to blind the endosonographer to the type of needle used, which may have introduced bias. However, this feature may not have been a significant limitation because the pathologists were blinded to the type of needle used for EUS-guided sampling.

## Conclusions

The 25G FNB needle was safe, reliably provided cytological diagnosis, and was comparable to the standard 22G FNA needle in patients with solid pancreatic masses in the absence of an on-site cytopathologist. The cytological sample quality in liquid-based preparation and the histological diagnostic yield for specific tumor discrimination of EUS-guided sampling using a 25G FNB needle were significantly higher than those using a 22G FNA needle.

## Abbreviations

EUS: Endoscopic ultrasound
G: Gauge
FNA: Fine needle aspiration
FNB: Fine needle biopsy
CS: Conventional smear
LBP: Liquid-based preparation
